# Impact of delisting OTC medicines from reimbursement list

**DOI:** 10.1186/2052-3211-8-S1-P4

**Published:** 2015-10-05

**Authors:** Petra Chytilová, Robin Šebesta

**Affiliations:** 1 State Institute for Drug Control (SIDC), Prague, 100 41, Czech Republic

## Background

This analysis addresses the impact of policy change when over-the-counter medicines (OTCs) were excluded from the reimbursement list by law since July 1, 2012 in the Czech Republic. Selected OTCs remained reimbursed with the approval of insurance funds, usually under certain conditions of reimbursement (for specific indications or patient groups).

## Objectives

This analysis aims at determining how many OTCs were reimbursed before the exclusion and how many of them and under what conditions of reimbursement remained to be reimbursed. The second objective of the analysis is to estimate the financial effect of exclusion on the payers and patients. The third objective is to describe what type of OTCs are reimbursed and under what conditions of reimbursement, as well as to compare annual costs of reimbursed OTCs versus annual costs of prescription-only medicines (POM).

## Methods

SIDC's list of reimbursed medicines valid as of 1 June 2012 was compared with the list of reimbursed medicines valid as of 1 July 2012 to identify OTCs excluded from reimbursement. The financial impact of delisting OTCs was calculated based on consumption data and reimbursement price from the payers. The current reimbursement list (May 2015) was searched to identify the current spectrum of reimbursed OTCs.

## Results

There were 238 reimbursed OTCs in June 2012 and 14 OTCs remained reimbursed in July 2012, based on registration number. The reimbursement is limited by conditions of reimbursement for most reimbursed OTCs. The delisted OTCs saved payers approximately 21.6 mil EUR (1 EUR = 27.624 CZK) in the first year after the delisting. The average price of not excluded OTCs has grown to 12.54% and the average price of excluded OTCs has grown to 10.70% since January 2012. The poster will show the analysis of consumption of OTCs in detail.

## Conclusions

Almost 95 % of previously reimbursed OTC medicines were excluded from the reimbursement system in 2012. OTC medicines that remain in the reimbursement system are reimbursed, for example, for patients with cystic fibrosis, diagnosed Sjogren's syndrome (dry eyes), chronic pancreatitis. The payers saved 21.6 mil. EUR due to the delisting. The delisting did not have a major impact on the price of excluded OTCs.

